# Statistical analysis plan for a cluster stepped-wedge randomized clinical trial assessing the effects of a multicomponent telemedicine-based intervention on quality of life in adults with respiratory failure requiring mechanical ventilation (Tele-Rehab MV Trial)

**DOI:** 10.62675/2965-2774.20260308

**Published:** 2026-02-20

**Authors:** Yasmin Ferreira Cavaliere, Rafael Barberena Moraes, Geraldine Trott, Maura Cristina dos Santos, Gabriela Soares Rech, Andrea de Carvalho, Ivan Ramos Maia, Fernando Godinho Zampieri, Adriano José Pereira, Regis Goulart Rosa

**Affiliations:** 1 Hospital Israelita Albert Einstein São Paulo SP Brazil Hospital Israelita Albert Einstein - São Paulo (SP), Brazil.; 2 Hospital Moinhos de Vento Porto Alegre RS Brazil Hospital Moinhos de Vento - Porto Alegre (RS), Brazil.

**Keywords:** Activities of daily living, Quality of life, Telemedicine, Return to work, Anxiety, Depression, Respiratory insufficiency, Patient discharge, Patient readmission, Quality improvement

## Abstract

**Objective:**

To describe the analytical objectives and procedures of the Tele-Rehab MV Trial prior to database lock.

**Methods:**

The Tele-Rehab MV Trial is a cluster stepped-wedge randomized clinical trial comparing a telemedicine-based quality improvement program focused on disability prevention and rehabilitation strategies with usual care. The intervention is implemented during the patient's intensive care unit stay, continued through ward admission, and extends up to 2 months post-hospital discharge. The trial targets adult patients with acute hypoxemic respiratory failure requiring invasive mechanical ventilation, in whom SARS-CoV-2 infection is part of the differential diagnosis. The protocol was approved by the Research Ethics Committee of the coordinating center and by the ethics committees of each of the 20 participating intensive care units, in accordance with Brazilian regulations. The primary outcome is health-related quality of life, assessed 90 days after hospital discharge using the EuroQol 5-Dimension 3-Level (EQ-5D-3L) scale. Secondary outcomes include 30-day rehospitalization, all-cause mortality, anxiety, depression, cognitive impairment, new disabilities in instrumental activities of daily living, and return to work or study 90 days after discharge. This report outlines the primary statistical procedures to be used for evaluating results and conducting sensitivity analyses.

**Conclusion:**

We anticipate that this reporting approach will minimize analysis bias and enhance the interpretation of the Tele-Rehab MV Trial results.

## INTRODUCTION

Acute hypoxemic respiratory failure requiring invasive mechanical ventilation (MV) is associated with high mortality and substantial long-term complications among survivors.^(1-3)^ These complications frequently include new physical limitations, cognitive decline, and psychological distress, all of which negatively impact the quality of life.^(3-5)^ Although care bundles - such as analgesia optimization, sedation minimization, early mobilization, *delirium* prevention, and screening for patients at risk of long-term disability to enable early rehabilitation and post-discharge care - have been recommended to mitigate these outcomes,^(5-7)^ no large-scale randomized trial has yet confirmed their effectiveness in improving long-term quality of life. In addition, logistical barriers frequently hinder patients’ attendance at in-person follow-up visits,^(8)^ underscoring telemedicine's potential to enhance access and reduce healthcare disparities.

The Tele-Rehab MV Trial aims to assess the impact of a multicomponent telemedicine-based intervention on the health-related quality of life of patients with acute hypoxemic respiratory failure requiring invasive MV, evaluated three months after hospital discharge. The present statistical analysis plan (SAP) aims to describe the trial's analytical objectives and procedures before locking the trial database, to enable analyses that comply with good clinical practice and avoid outcome reporting bias.

### Trial overview

The study background, design, rationale, eligibility criteria, and sample size have been previously published.^(9)^ Briefly, this is a stepped-wedge cluster randomized controlled trial designed to evaluate a multicomponent telemedicine-based intervention aimed at improving post-intensive care unit (ICU) recovery in patients with acute hypoxemic respiratory failure (NCT06343545). The unit of randomization is the ICU, with sequential transitions from control (standard of care) to intervention every 2 months until all participating clusters are exposed to the intervention. Randomization is stratified by the number of ICU beds and conducted by a statistician who is not involved in study implementation or recruitment. The randomization list is securely stored and accessible only to the statistician. Intensive care unit assignments are disclosed to local teams 60 days before implementation to allow adequate time for training, but teams maintain usual care practices until the official start of the intervention. Due to the nature of the intervention and study design, blinding of ICU staff and participants is not feasible. Participants are monitored daily during hospitalization by trained site researchers. After rehospitalization, blinded researchers conduct centralized telephone interviews at 15, 30, 60, and 90 days to assess outcomes.

Clusters include Brazilian public hospital ICUs with at least eight beds and the capacity for remote care. Patient-level inclusion criteria are adults (≥ 18 years) with hypoxemic acute respiratory failure requiring invasive MV, in whom severe acute respiratory syndrome coronavirus 2 (SARS-CoV-2) infection is part of the differential diagnosis. This does not imply that coronavirus disease 2019 (COVID-19) is the primary suspected cause, but rather that it is considered at least a possible (though not necessarily probable) diagnosis at ICU admission. Accordingly, a positive SARS-CoV-2 test will not be required for inclusion.

The intervention consists of three telemedicine-based bundles: the ICU tele-bundle, the ward tele-bundle, and the post-discharge tele-bundle. In the ICU tele-bundle, trained local staff apply evidence-based strategies during daily rounds, including analgesia optimization, sedation minimization, spontaneous breathing trials, *delirium* prevention, early mobilization, and removal of unnecessary invasive devices, with remote support from critical care specialists via telemedicine. In the ward tele-bundle, local teams or centralized tele-rehabilitation specialists screen patients at high risk for post-ICU disabilities and develop personalized rehabilitation plans to be initiated during ward hospitalization and continued after discharge. After discharge, a 2-month tele-rehabilitation program (post-discharge tele-bundle) is delivered via videoconferencing, including physical, respiratory, swallowing, and psychological therapies. Nurse navigators ensure continuity of care by facilitating multidisciplinary communication across all phases and supporting adherence to the intervention.

The primary outcome is health-related quality of life at 90 days post-discharge, assessed using the EuroQol five-dimension three-level (EQ-5D-3L).^(10)^ Secondary outcomes include 30-day hospital readmission, all-cause mortality, and clinical status within 90 days, and at 90 days - anxiety and depression symptoms, cognitive impairment, new disabilities in instrumental activities of daily living, physical dependence, EQ-5D-3L utility scores among survivors, and return to work or studies.

### Statistical analysis plan

#### Overall principles

The primary analysis for each outcome will be conducted at the individual participant level. All analyses will account for the cluster stepped-wedge design to ensure appropriate control of type I error, with outcomes analyzed according to the randomized group assignment. A two-sided significance level of 0.05 will be used for all statistical comparisons. Effect sizes will be reported as adjusted risk differences and risk ratios or odds ratios for binary outcomes, mean differences for continuous outcomes, and odds ratios for ordinal outcomes, all presented with 95% confidence intervals. All models will report estimates of the intracluster correlation coefficient (ICC) to quantify between-cluster variance. Secondary outcomes and subgroup analyses will not be adjusted for multiple comparisons and will therefore be interpreted as exploratory. Analyses will commence after completion of follow-up, database cleaning, and locking, and submission of the SAP for publication. All analyses will be performed using R (R Development Core Team)^(11)^ in a version to be specified at the time of data analysis.

#### Handling of missing data

Missing values for the EQ-5D-3L utility scores and covariates used in model adjustment will be handled using multiple imputation by chained equations (MICE)^(12)^ if the missingness rate exceeds 5%. We will generate 25 imputed datasets using the mice package in R, with the imputation model including all outcome variables, baseline covariates (age, sex, Charlson Comorbidity Index [CCI], Simplified Acute Physiology Score III [SAPS III]), cluster identifier, and time period to preserve the hierarchical structure and temporal correlation inherent in the stepped-wedge design. The imputation model will use predictive mean matching for continuous variables and logistic regression for binary variables. Convergence will be assessed by visual inspection of trace plots and the potential scale reduction factor. Results from the imputed datasets will be pooled using Rubin's rules to obtain final parameter estimates and standard errors. The missing at random assumption will be evaluated by comparing baseline characteristics between participants with and without missing outcome data. If the proportion of missing data is ≤ 5%, analyses will be conducted using only complete case data.

#### Definition of analysis sets

At the cluster level, the primary analysis set will include all randomized ICUs that enrolled participants, regardless of adherence to the study interventions or the number of participants enrolled. At the individual participant level, the primary analysis set will include all enrolled participants, except those who were transferred to another hospital during their ICU or ward stay, and those for whom the patient or proxy did not provide consent to participate. A secondary analysis set at the individual participant level will include all enrolled participants, excluding only those without consent to participate; this set will be used for sensitivity analyses.

### Statistical analyses

#### Patient flow

The flow of participants will be displayed in accordance with the Consolidated Standards of Reporting Trials (CONSORT) flow diagram (CONSORT 2010 statement: extension to cluster-randomized trials).^(13)^ This description will include information about eligibility criteria and follow-up losses at both cluster and subject levels.

#### Adherence to study interventions

To evaluate the intervention's fidelity, weekly remote monitoring sessions will be conducted across all participating clusters. During these sessions, researchers from the coordinating center will conduct semi-structured interviews with ICU and ward staff to assess adherence to the intervention's core components. As an additional adherence indicator, rates of patient engagement in the tele-rehabilitation program will also be tracked. Throughout the intervention phase, each cluster will undergo structured assessments, with adherence scored on a scale from zero to 100%, where higher scores reflect stronger implementation of the intervention. Adherence will be examined across three distinct domains: ICU tele-bundle, encompassing multidisciplinary rounds, optimization of analgesia, reduction of sedation, spontaneous breathing trials, *delirium* prevention strategies, early mobilization, and removal of unnecessary invasive devices; ward tele-bundle, which includes the identification of patients at elevated risk for post-ICU complications and the initiation of individualized rehabilitation plans during hospitalization on the ward; and post-discharge tele-bundle, measured by patient attendance at scheduled teleconsultations. A composite adherence score will be calculated as the median of the scores across all three domains.

#### Baseline characteristics

Baseline characteristics of all participants will be presented by study arm in tabular form. No formal hypothesis testing will be conducted to avoid unnecessary statistical comparisons. Categorical variables will be reported as absolute and relative frequencies. In contrast, continuous or discrete variables will be summarized using measures of central tendency (mean or median) and dispersion (standard deviation or interquartile range), as appropriate.

#### Primary outcome

The primary outcome is the utility index derived from the EQ-5D-3L,^(10)^ assessed 90 days after hospital discharge. Patients who die before this time point will be assigned a utility score of zero. The index, based on the Brazilian population value set, ranges from −0.17 to one, where zero represents a health state equivalent to death, negative values represent states considered worse than death, and one corresponds to full health.^(14)^ Comparisons between study arms will be performed using a generalized linear mixed model (GLMM), with ICU treated as a random effect and time as a fixed effect, following the approach described by Hussey and Hughes.^(15)^ The model will be adjusted for the following individual-level covariates: age, sex, CCI score,^(16)^ and SAPS III score.^(17)^ Potential non-linear effects of continuous individual-level covariates (age, CCI score, and SAPS III score) will be evaluated using restricted cubic splines or fractional polynomials, with retention of non-linear terms guided by graphical assessment and model fit criteria. The model matrix includes fixed effects for the intervention period (pre- *versus* post-implementation) and fixed individual-level covariates. The general specification of the model is:


$$\begin{array}{c} g\left(\mu_{ijt}\right)=\beta_{0}+\beta_{1}\;Intervention{\;}_{jt}+\beta_{2}\;Age{\;}_{ijt}+\beta_{3}\;Sex{\;}_{ijt}+\\ \beta_{4}\;CCI\;score{\;}_{ijt}+\beta_{5}\;SAPS\;III\;score{\;}_{ijt}+u_{j}+v_{t} \end{array}$$

where,

μ_ijt_: is the expected value of the outcome for an individual in a cluster at time.

is the link function appropriate to the outcome (e.g., logit for binary, identity for continuous).

β_0_ is the intercept.

β_1_ represents the effect of the intervention (leading coefficient of interest).

*u*_j_ ~ *N* (0,σ^2^_u_) is the random effect for the cluster (hospital).

ν_t_ ~ *N* (0,σ^2^ν) is the fixed effect for time (period).

For both primary and secondary outcomes, the link function in the GLMM will be selected based on the outcome type: identity for continuous outcomes, logit for binary outcomes, log for count outcomes, and cumulative logit for ordinal outcomes.

#### Sensitivity analyses for the primary outcome

We aim to conduct the following sensitivity analyses for the primary outcome to assess consistency and the risk of bias:

1. Estimation of the intervention effect using the same model as in the primary analysis, stratified by quartiles of the overall cluster adherence score.
2. Estimation of the intervention effect using the same model as in the primary analysis, stratified by quartiles of adherence scores within each domain (ICU tele-bundle, ward tele-bundle, and post-discharge tele-bundle).
3. Estimation of the intervention effect in the dataset that includes patients who were transferred to another hospital during their ICU or ward stay (secondary analysis set).
4. Estimation of the intervention effect using zero-inflated models, such as zero-inflated Poisson (ZIP) or zero-inflated negative binomial (ZINB), depending on the observed dispersion. The zero-inflation component will model excess zeros independently of the count process. Results from these models will be compared to those from the main GLMMs to assess the robustness of the findings. Model selection will be guided by goodness-of-fit criteria and diagnostic measures (e.g., AIC, BIC, residual plots).

#### Subgroup analyses for the primary outcome

There will be five a priori defined subgroup analyses for the primary outcome: age (< 65 years *versus* ≥ 65 years), sex, SAPS-3 score (< *versus* ≥ median), pre-admission Barthel index (< 90 *versus* ≥ 90), and COVID-19 *versus* other aetiologies of acute respiratory failure. The consistency of intervention effects across the aforementioned subgroups will be assessed using tests for interaction. In addition to analyses based on dichotomization or quartiles of continuous variables, we will assess non-linear interaction terms to better capture potential effect modification in secondary subgroup analyses.

#### Secondary outcomes

We will use the following statistical procedures to evaluate the study's secondary outcomes in the primary analysis set:

1. **Rehospitalization within 30 days after hospital discharge:** the differences between the study arms will be compared using a GLMM with with ICU treated as a random effect and time as a fixed effect. The model will be adjusted by the following individual-level covariates: age, sex, CCI score, and SAPS III score.
2. **All-cause mortality within 90 days of enrollment:** differences between study arms will be compared using a GLMM with with ICU treated as a random effect and time as a fixed effect. The model will be adjusted by the following individual-level covariates: age, sex, CCI score, and SAPS III score.
3. **Clinical status within 90 days:** assessed according to a modified version of the World Health Organization Ordinal Scale for Clinical Improvement (scores range from 1 [best] to 8 [worse]) during hospitalization and at 15-, 30-, 60-, and 90-day post-discharge.^(9)^ The differences between study arms will be compared using the Markov Longitudinal Ordinal Model. The model will be adjusted by the following individual-level covariates: age, sex, CCI score, and SAPS III score.
4. **Days alive and free of hospital at 90 days:** Patients who die before day 90 are assigned a score of zero. Differences between study arms will be compared using GLMM, with ICU treated as a random effect and time as a fixed effect. The model will be adjusted by the following individual-level covariates: age, sex, CCI score, and SAPS III score.
5. **Symptoms of anxiety and depression 90 days after hospital discharge:** assessed by the Hospital Anxiety and Depression Scale (with scores > 7 and > 10 indicating possible and probable cases of anxiety or depression, respectively).^(18)^ The differences in the prevalence of possible and probable anxiety and depression between study arms will be compared using GLMM with ICU treated as a random effect and time as a fixed effect. The model will be adjusted by the following individual-level covariates: age, sex, CCI score, SAPS III score, and previous history of anxiety or depression.
6. **Cognitive impairment 90 days after hospital discharge:** assessed by the modified Telephone Interview for Cognitive Status (with scores < 14 indicating cognitive impairment).^(19)^ The differences in the prevalence of cognitive impairment between study arms will be compared using logistic regression with ICU treated as a random effect and time as a fixed effect. The model will be adjusted by the following individual-level covariates: age, sex, CCI score, and SAPS III score.
7. **New disabilities in instrumental activities of daily living 90 days after hospital discharge:** assessed by the Lawton and Brody instrumental activities of daily living scale (any impairment, moving from independent to partially dependent or from partially dependent to totally dependent, in at least one of the following domains: telephone use, transportation, shopping, responsibility for own medications, and ability to handle finances) relative to 1 month before hospitalization.^(20)^ The differences between study arms will be compared using GLMM with ICU treated as a random effect and time as a fixed effect. The model will be adjusted by the following individual-level covariates: age, sex, Charlson CCI score, and SAPS III score.
8. **Physical dependence 90 days after hospital discharge:** assessed by the modified Barthel index (with scores < 21, 21 - 60, and 61 - 90, indicating total, severe, and moderate dependence, respectively).^(21)^ The differences in the prevalence of total, severe, and moderate dependence between study arms will be compared using GLMM with ICU treated as a random effect and time as a fixed effect. The model will be adjusted by the following individual-level covariates: age, sex, CCI, and SAPS III.
9. **EQ-5D-3L utility scores 90 days after hospital discharge among survivors:** differences between study arms will be compared using a GLMM with ICU treated as a random effect and time as a fixed effect. The model will be adjusted by the following individual-level covariates: age, sex, CCI score, and SAPS III score.
10. **Return to work or studies within 90 days of hospital discharge:** applicable to patients who were employed or enrolled in education at the time of hospital admission. The differences between study arms will be compared using GLMM with ICU treated as a random effect and time as a fixed effect. The model will be adjusted by the following individual-level covariates: age, sex, CCI score, and SAPS III score.

## DISCUSSION AND TRIAL STATUS

In this SAP, we present the statistical procedures to assess the effectiveness of implementing a telemedicine-based quality improvement program focused on disability prevention and rehabilitation strategies. The present publication aims to avoid risks of outcome reporting bias and data-driven results, and to provide guidance on statistical analysis for future studies in this field. As of August 2025, 20 ICUs were enrolled.

## Data Availability

The data generated and/or analyzed during the current study will be available from the corresponding author upon reasonable request. Data sharing will be subject to approval by the study Steering Committee and in accordance with applicable ethical and regulatory requirements, including participant confidentiality and data protection regulations.
